# Group cognitive behavioral therapy modulates the resting-state functional connectivity of amygdala-related network in patients with generalized social anxiety disorder

**DOI:** 10.1186/s12888-016-0904-8

**Published:** 2016-06-13

**Authors:** Minlan Yuan, Hongru Zhu, Changjian Qiu, Yajing Meng, Yan Zhang, Jing Shang, Xiaojing Nie, Zhengjia Ren, Qiyong Gong, Wei Zhang, Su Lui

**Affiliations:** Mental Health Center, West China Hospital of Sichuan University, No. 37 Guo Xue Xiang, Chengdu, 610041 China; State Key Laboratory of Biotherapy, Psychiatric Laboratory, West China Hospital of Sichuan University, Chengdu, Sichuan China; Department of Radiology, Huaxi MR Research Center (HMRRC), West China Hospital of Sichuan University, No. 37 Guo Xue Xiang, Chengdu, 610041 China; Radiology Department, the Second Affiliated Hospital, Wenzhou Medical University, Wenzhou, Zhejiang China

**Keywords:** Generalized social anxiety disorder, Group cognitive–behavioral therapy, Functional magnetic resonance imaging, Resting-state, Brain network

## Abstract

**Background:**

Amygdala is considered as the core pathogenesis of generalized social anxiety disorder (GSAD). However, it is still unclear whether effective group cognitive behavioral therapy (CBT) could modulate the function of amygdala-related network. We aimed to examine the resting-state functional connectivity (rsFC) of the amygdala before and after group CBT.

**Methods:**

Fifteen patients with GSAD were scanned on a 3T MR system before and after 8 weeks of group CBT. For comparison, nineteen healthy control participants also underwent baseline fMRI scanning. We used bilateral amygdala as seed regions and the rsFC maps of the right and left amygdala were created separately in a voxel-wise way. Clusters survived two-tailed Gaussian Random Field (GRF) correction at p <0.05 (voxel z value >2.3).

**Results:**

Compared with baseline, patients with CBT showed significantly decreased connectivity of the left amygdala with the right putamen, the left dorsal medial prefrontal cortex (dmPFC) and the right dorsal anterior cingulate cortex (dACC). Especially, the changes of the connectivity between the left amygdala and the dACC positively correlated with changes of the anxiety symptom in patients. Furthermore, in relative to controls, patients showed higher connectivity of left amygdala with dmPFC and dACC at baseline, while normal after CBT.

**Conclusions:**

Short-term group CBT could down-regulate the abnormal higher connectivity of prefrontal-amygdala network, along with clinical improvement. This may provide a potential biomarker to monitor the treatment effect of CBT in GSAD patients.

## Background

Generalized social anxiety disorder (GSAD) is a common mental disorder that mainly involves a notable fear and avoidance of most social or performance situations, [1] and has a point prevalence of 4.4 % [2]. The repeated experience of anxiety in relatively harmless situations interferes with substantial occupational performance and relationships of GSAD patients [3]. Evidences from animal models [4, 5] and human beings [6, 7] have indicated the role of amygdala as the core in the pathogenesis of GSAD. Especially, neuroimaging studies have detected decreased volume of the amygdala [8] and increased functional activity of the amygdala in response to aversive and threatening social stimuli in GSAD patients [9–11]. Further studies began to study the interaction of amygdala with other brain regions since the GSAD are attributable to the mis-communication among different brain regions in a wide network rather than a single specific brain structure [12]. Impaired connectivity between amygdala and anterior cingulate cortex (ACC) rather than the activity pattern of the amygdala has been found to be related to anxiety symptoms severity [13]. Furthermore, anxiety was associated with a negative connectivity between amygdala and ventral medial prefrontal cortex (mPFC), suggesting disrupted emotion regulation. Besides, a positive connectivity between amygdala and dorsal mPFC was also related to anxiety, indicating hypervigilance to external stimuli [14]. Abnormal amygdala connectivity with other brain regions such as precuneus [15] and insula [16] in GSAD have also been revealed.

However, another question raises that whether these aberrant amygdala-related networks can be modulated along with clinical improvement after treatment. Investigation of the brain changes before and after treatment not only improves our understanding of the pathogenesis and recovery of GSAD, but can also help to monitor treatment effects and guide the selection of the optimal therapy [17]. The resting-state functional connectivity (rsFC) method has become a valuable tool for investigating network function, as it allows for a paradigm-free analysis of connectivity in fMRI without a priori assumptions about neural activation [18, 19]. Besides, the potentially powerful method enables us to increase our understanding and develop a more refined and comprehensive brain modulatory model of GSAD before and after treatment.

Using rsFC, some studies found the neuropeptide oxytocin could affect the rsFC of the amygdala with rostral ACC/ventral mPFC in GSAD patients [20]. However, as another important treatment method [21], cognitive–behavioral therapy (CBT) appears to be equally effective but more enduring [22, 23] and safer, with fewer side effects. A recent study also showed that the resting-state amygdala-prefrontal connectivity at baseline could predict the prognosis of GSAD patients after CBT [24]. It is still unclear that whether the standardized group CBT [25] could modulate amygdala-related functional connectivity along with clinical improvement. Based on the literature, we hypothesized that certain amygdala-related neural network was disrupted compared to healthy control subjects at baseline, which would be normalized by group CBT along with clinical improvement.

## Methods

### Participants

Individuals with GSAD were enrolled by referrals from the outpatient clinic of Mental Health Center, West China Hospital, Chengdu, China from March 2011 to December 2013. Eighteen patients completed a baseline fMRI scan, 8 weeks of group CBT, and a second fMRI scan after treatment. One female and two male patients were excluded due to the sever head motion during the MRI scan. Thus, 15 patients (10 males; mean age, 27.07 ± 8.11 years) were included in the final statistical analysis. For comparison, we enrolled a group of 19 demographically matched healthy controls (13 males; mean age, 26.26 ± 4.90 years), who were volunteers from the community. Table 1 shows all the participants’ demographic and clinical characteristics. Four patients were on a stable dosage of a selective serotonin reuptake inhibitor for at least 4 weeks (two patients were treated with paroxetine, 20 mg/day, one patient was treated with paroxetine, 10 mg/day, and one patient was treated with paroxetine, 20 mg/day as well as tandospirone, 20 mg/day), but had had to discontinue psychotropic medication due to poor response at least 2 weeks prior to the baseline MRI scan.

**Table 1:** Demographic information and psychological variables in GSAD and HC groups before and after treatment

	HC (n = 19)	Pretreatment GSAD (*n* = 15)	Posttreatment GSAD (*n* = 15)	T (pre-treatment vs HCs)	P (pre-treatment vs HCs)	T (pretreatment vs posttreatment)	P (pretreatment vs posttreatment)
Age (Years)	26.26 ± 4.90	27.07 ± 8.11	_	0.36	0.723	_	_
Education (Years)	15.95 ± 2.82	11.27 ± 3.35	_	−4.43	<0.001	_	_
Total LSAS (mean ± SD)	24.53 ± 17.23	78.87 ± 27.00	50.93 ± 19.13	7.14	<0.001	5.80	<0.001
LSAS _fear_ (mean ± SD)	11.00 ± 9.68	38.13 ± 13.21	26.60 ± 8.80	6.92	<0.001	5.50	<0.001
LSAS _avoid_ (mean ± SD)	13.53 ± 10.85	40.73 ± 15.22	24.33 ± 12.75	6.08	<0.001	5.11	<0.001
HAMA (mean ± SD)	2.21 ± 2.64	14.53 ± 8.51	9.33 ± 5.27	5.98	<0.001	2.59	0.021
HAMD (mean ± SD)	2.11 ± 2.62	11.00 ± 6.75	7.00 ± 4.58	5.28	<0.001	3.30	0.005

*GSAD* generalized social anxiety disorder, *HC* healthy control, *LSAS* Liebowitz Social Anxiety Scale, *HAMD* Hamilton rating scale for depression, *HAMA* Hamilton rating scale for anxiety

### Psychometric measures

The psychiatric diagnostic classification of the participants was based on the Structured Clinical Interview for DSM-IV axis I disorders (SCID) [26] by psychiatrists. All participants were right-handed. The participants were interviewed to confirm that there was no history of psychiatric illness among their first-degree relatives, and that they themselves had no history of major medical or neurologic illness, and no history of substance abuse within a 6-month period prior to scanning. The patients had to fulfill the criteria for GSAD according to the SCID. The exclusion criteria for the patient group included current psychotherapy, past CBT, and other DSM-IV diagnoses, thus the patients included had no comorbidty.

Symptom severity was assessed using the Liebowitz Social Anxiety Scale Self-Report [27, 28] (LSAS-SR) at the time of inclusion in the study and after treatment. The Hamilton Anxiety Rating Scale (HAMA) and the Hamilton Depression Rating Scale (HAMD) were also used to measure anxiety and depression for all the participants.

### Treatment phase

Patients participated in 8 weekly CBT sessions in a group format according to the principles described by Hope, Heimberg [29] and Hofmann [25]. The therapy consisted of one 150-min session per week, according to a standardized protocol-based group treatment [25]. The sessions were conducted by a licensed clinical psychologist with several years of working experience in group CBT. A licensed clinical psychologist with expertise in both CBT and clinical trial studies involving CBT supervised the therapy to ensure adherence to treatment. The two psychologists were not involved in the subsequent analysis of treatment outcome and fMRI data. The group CBT comprised of psychoeducation, cognitive restructuring, relaxation training, in vivo exposures to public speaking, social skills training and homework assignment.

### Functional MRI scan acquisition

A 3.0 T magnetic resonance scanner (Siemens 3.0 T Trio Tim, Germany) with a twelve-channel phased-array head coil at the Huaxi MR Research Center was used. Each participant was positioned supine in the MRI scanner with foam padding to reduce head movements. Functional images were acquired using a single-shot, gradient-recalled echo-planar imaging sequence (repetition time, 2,000 ms; echo time, 30 ms; flip angle, 90°; field of view, 240 mm × 240 mm; matrix, 64 × 64; thickness, 5 mm; without gap), which yielded a voxel size of 3.75 mm × 3.75 mm × 5 mm. Each brain volume comprised of 30 axial slices and each functional run contained 205 image volumes. Participants were instructed to rest with their eyes closed and let their minds wander but not fall asleep. The scan lasted 6.8 min.

### Functional MRI data preprocessing

Imaging preprocessing was performed with Data Processing Assistant for Resting-State fMRI Basic Edition (DPARSF_3.0; http://rfmri.org/DPARSF). All software programs were run on a Statistical Parametric Mapping platform, (SPM8, http://www.fil.ion.ucl.ac.uk/spm), based on the MATLAB programming language (The Mathworks Inc, USA). The first 10 volumes from each run were discarded to ensure the stability of the imaging data. In the first step, the functional images were corrected for slice timing, and participants with head motion of more than 3.0 mm in the x, y, or z direction and more than 1.0° of rotation about each axis were excluded. Next, the realigned images were spatially normalized to the EPI template in SPM8, resampled to 3 mm cubic voxels, and smoothed by convolution with an isotropic Gaussian kernel (full width half maximum, 4 mm) to decrease spatial noise. Linear trend and quadratic trend entered in the regression analysis. Cerebrospinal fluid, white matter, 6 head motion parameters, 6 head motion parameters one time point before, and the 12 corresponding squared items were removed from the images [30]. Considering the increasing evidence that global brain signal regression could alter inter-individual differences [31], the global signal regression was not performed. The resting data were bandpass-filtered between frequencies of 0.01 and 0.1 to limit the analysis to the resting-state frequencies of interest.

### Functional MRI data analysis

By applying a seed-region approach [32], we used the right and left amygdala seeds, defined within the automated anatomical labeling (AAL) [33] atlas using MarsBar toolbox (http://marsbar.sourceforge.net) for FC analyses. The resulting r values were converted to z-values using Fisher's r-to-z transformation to improve the Gaussianity of their distribution. The FC maps of both the right and left amygdala were created using the resting-state fMRI data processing toolkit (REST V1.8, State Key Laboratory of Cognitive Neuroscience and Learning in Beijing Normal University; 〈http://www.restfmri.net/forum/REST_V1.8〉).

We then performed several statistical parametric tests in REST to test our priori hypotheses. To examine the changed amygdala-related network compared to the baseline status after group CBT, the FC maps in the pre-treatment and post-treatment groups were compared on a voxel-wise basis via a paired sample t-test. The Gaussian Random Field (GRF) theory correction was used for the statistical significance of group differences, with a voxel z threshold of a voxel p value <0.0214, a cluster p value <0.1 (two tailed), and a cluster connectivity of 5 mm, which is equivalent to voxel z value >2.3, cluster *p* value < 0.05 (two one tail corrections) in the SPM. Then, we defined regions of interest (ROI) based on functional clusters showing significant differences between pre-treatment and post-treatment groups. These seed regions were used as masks to test whether the pre- versus post-treatment findings in patients were different between patients and healthy controls (HC). Two sample t-test was used to characterize the difference between patients and controls (*p* < 0.05, voxel z value > 2.3, GRF corrected). Cluster size was set at least 5 voxels (135 mm^3^), with cluster connectivity of 5 mm. Gender, age, sex, education years and frame-wise displacement (FD) measurements of head motion [34] were modeled as covariates to remove their impacts.

After multiple comparison corrections, we further identified the surviving clusters by comparison with the neuroanatomical atlas by visual assessment and cross-referenced reports. Finally, the FC values of the seed regions and functional clusters within the seed regions that survived the voxel correction criteria between pre-treatment and HC groups were extracted by REST toolkit and used for further analysis. To assess for correlations with symptom severity, the extracted FC values from the GSAD pre-treatment scans were correlated with the LSAS (including total score, fear factor and avoidance factor) and HAMA using Pearson correlation analysis. Pearson correlation coefficient analysis was also conducted between the degree of change in the extracted FC values (Δ_FC/PreFC_) and the scale of improvement in LSAS (Δ_LSAS/PreLSAS_) and HAMA (Δ_HAMA/PreHAMA_) scores upon treatment in order to test the hypothesis that FC changes would parallel the clinical response to treatment. (SPSS version 19, IBM, USA).

## Results

### Treatment results

After 8 weeks of group CBT, the total and the subscale scores of LSAS-SR significantly decreased (*p* < 0.05) in patients group, with the average total scores reduced from 78.87 ± 27.00 to 50.93 ± 19.13. Also, the HAMA scores reduced from an average of 14.53 ± 8.51 to 9.33 ± 5.27 (*p* < 0.05), while HAMA decreased from an average of 11.00 ± 6.75 to 7.00 ± 4.58 (*p* < 0.05). These findings indicate the CBT is effective in treatment of the symptoms of GSAD patients (Table 1).

### Connectivity analysis

**Fig. 1:**
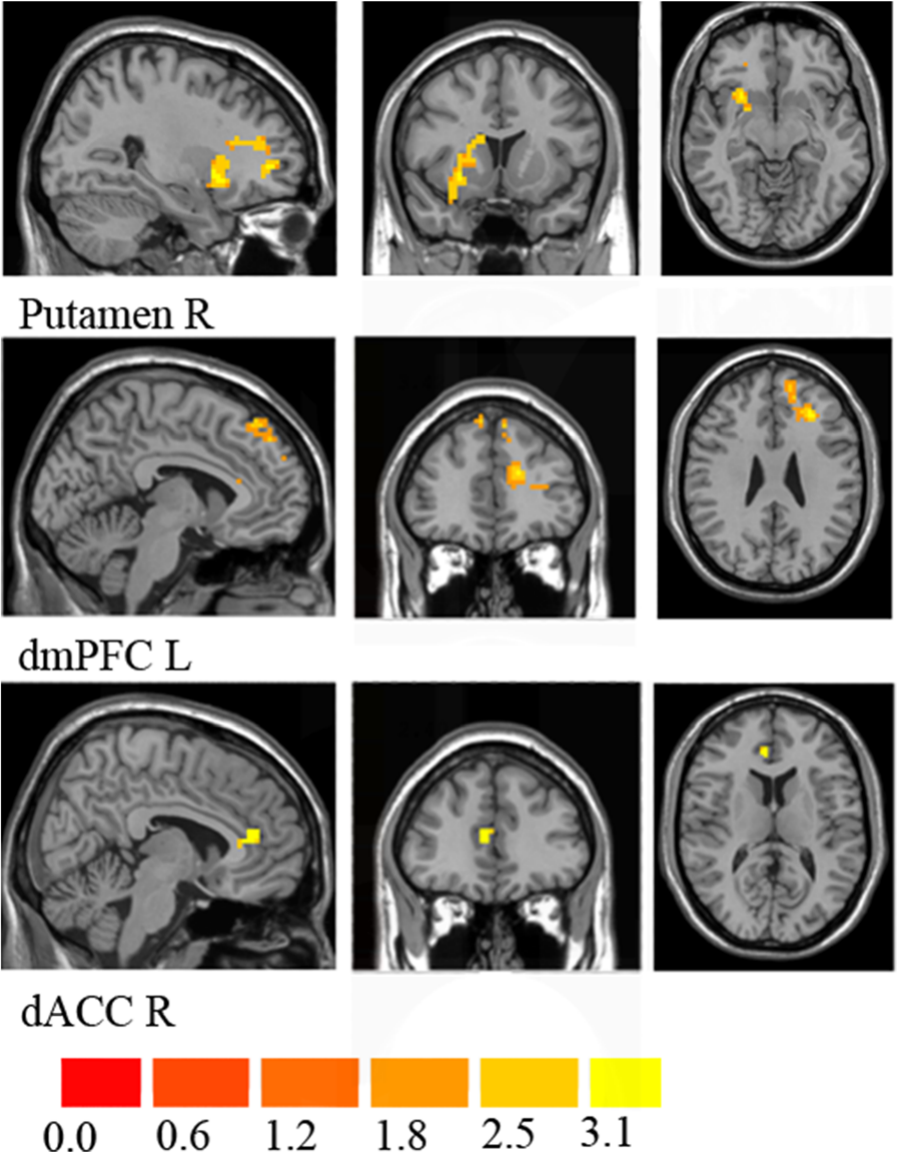
Statistical t-map of resting-state functional connectivity of amygdala between the pre-treatment and post-treatment groups. Compared to the baseline, patients with GSAD showed decreased connectivity of the left amygdala with the right putamen, the left dmPFC and the right dACC. (paired sample t-test, p <0.05, voxel z value >2.3, GRF corrected, two-tailed). No differences were found for the right amygdala connectivity between the pre-treatment and post-treatment groups. dmPFC, dorsal medial prefrontal cortex; dACC, dorsal anterior cingulate cortex; L, left; R, right

**Fig. 2:**
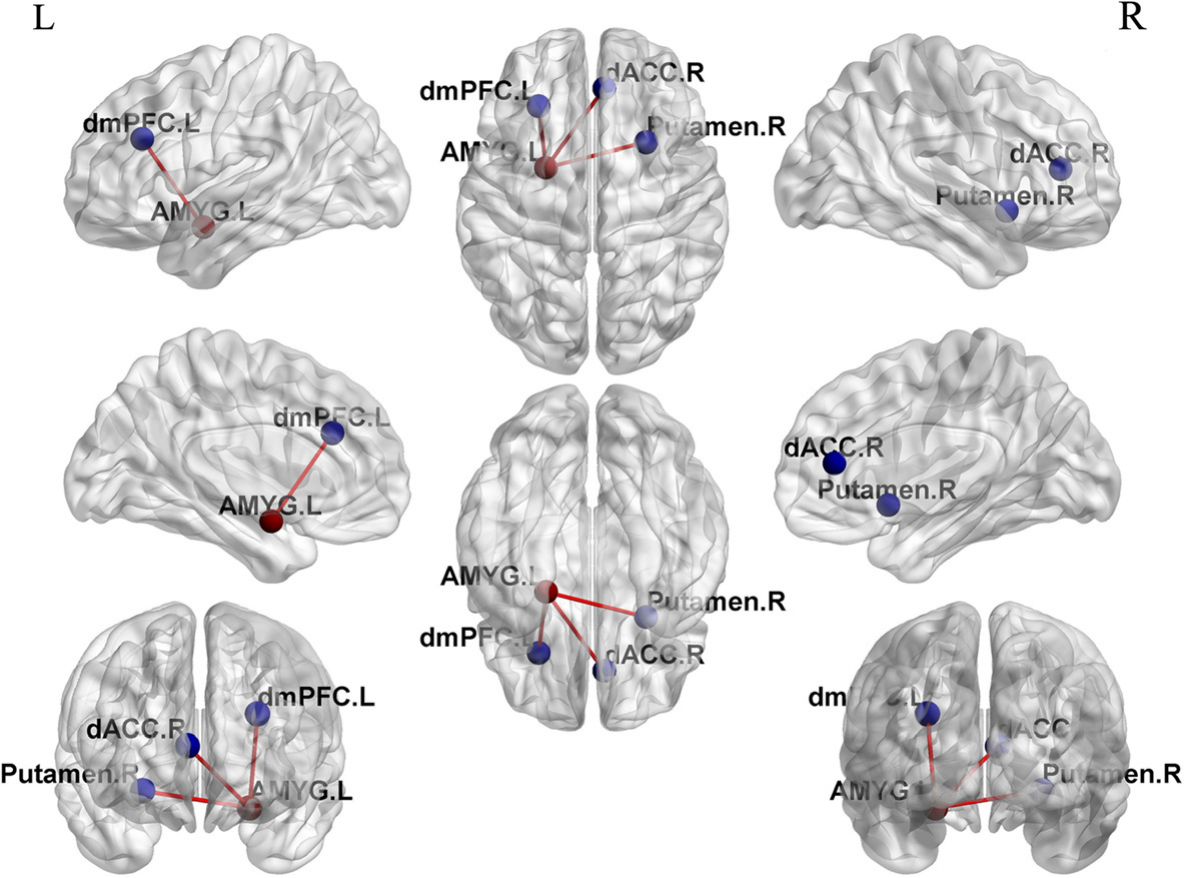
Statistically significant differences in resting-state functional connectivity of amygdala between the pre-treatment and post-treatment groups. Compared to the baseline, patients with GSAD showed decreased connectivity of the left amygdala with the right putamen, the left dmPFC and the right dACC. (paired sample t-test, *p* <0.05, voxel z value >2.3, GRF corrected, two-tailed). The left amygdala seed was mapped onto the brain regions at three views: from left to right, from front to back and from right to left. No differences were found for the right amygdala connectivity between the pre-treatment and post-treatment groups. dmPFC, dorsal medial prefrontal cortex; dACC, dorsal anterior cingulate cortex; L, left; R, right

Compared to the baseline, patients with GSAD showed decreased connectivity of the left amygdala with three brain regions: the right putamen, the left dorsal portions of the PFC (dmPFC), and the right dorsal ACC (dACC) (Figs. 1 and 2). No differences were found for the right amygdala connectivity between the pre-treatment and post-treatment groups. The changes of extracted FC values (Δ_FC/PreFC_) in left amygdala-right ACC were positively correlated with changes of the HAMA scores (Δ_HAMA/PreHAMA_) (*r* = 0.823, *p* <0.001). No significant correlation of extracted FC values from the GSAD pre-treatment scans and symptom severity was revealed.

**Fig. 3:**
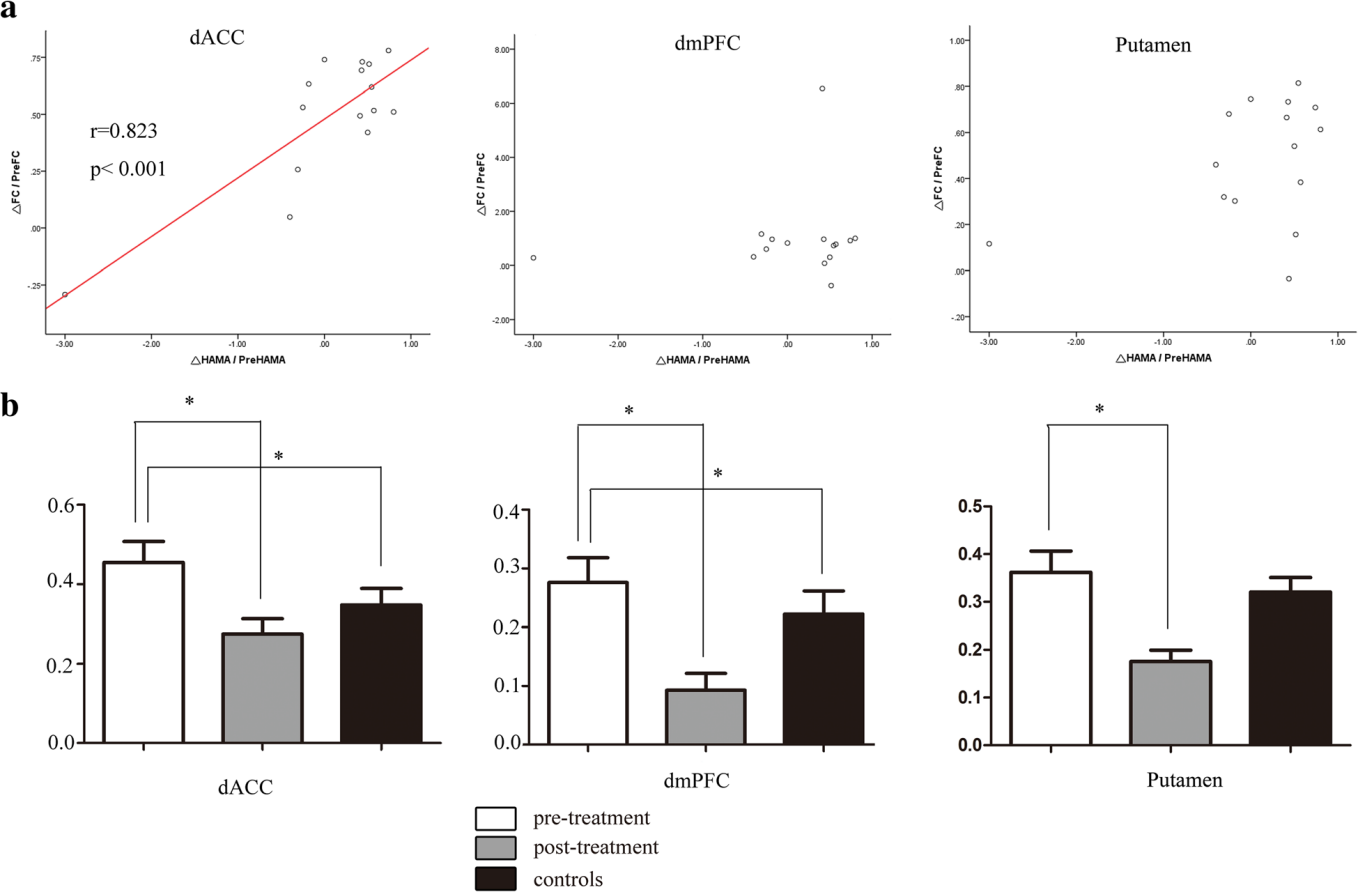
**a** Scatter plots showing significant correlation between the degree of change to mean rsFC and the scale of improvement in HAMA scores in dACC (*p* <0.001). The preHAMA and preFC in dACC decreased after CBT, and changes of FC paralleled symptom improvements. When removing the outlier on the left of the scatter plots, there are still significant correlations (*p* <0.05). **b** Mean rsFC value of each altered region. Pre, pre-treatment; rsFC, resting-state functional connectivity; HAMA, Hamilton rating scale for anxiety; dACC, dorsal anterior cingulate cortex; dmPFC, dorsal medial prefrontal cortex. * Comparison is significant at the 0.05 level (GRF corrected)

To test whether the connectivity of the left amygdala with above three brain regions were different between the patients and controls, the survived three clusters were used as masks for group comparisons of the amygdala related connectivity maps between patients and controls. In relative to controls, the patient group showed higher connectivity of the left amygdala with the right dACC and the left dmPFC at baseline, but recovered to normal after CBT. (Fig. 3, Table 2).

**Table 2:** Altered amygdala connectivity with other brain regions

Contrast	Brain region	Brodmann area	MNI Coordinate			T score	Voxels
			x	y	z		
Altered amygdala connectivity in GSAD patients before and after treatment							
GSAD Pre > GSAD Post							
Amygdala L	Putamen R	_	27	12	−9	3.67^a^	392
	dmPFC L	46	−27	30	27	4.10^a^	329
	dACC R	32	6	39	12	2.88^a^	19
Amygdala R	none						
Abnormal amygdala connectivity in pre-treatment group compared with HC GSAD Pre > HC							
Amygdala L	dmPFC L	46	−30	36	9	2.62^b^	11
	dACC R	32	6	39	9	2.76^b^	7
Abnormal amygdala connectivity in post-treatment group compared with HC							
none							

No significant voxels for GSAD Pre < GSAD Post or GSAD Pre < HC

*p* <0.05, voxel z value >2.3, Gaussian Random Field (GRF) corrected (two-tailed); *MNI* Montreal Neurologic Institute, *GSAD* generalized social anxiety disorder (entire cohort, *N* = 15), *HC* healthy control (*N* = 19), *Pre* pretreatment, *Post* post-treatment, *dmPFC* dorsal medial prefrontal cortex, *dACC* dorsal medial anterior cingulate cortex, *L* left, *R* right, *vs* versus; a: paired sample T-test; b: two sample T-test

## Discussion

In this longitudinal study, we used fMRI to investigate changes of rsFC in patients with GSAD before and after 8 weekly group CBT treatment. Based on a standard clinician measure, the majority of patients improved following treatment. As we hypothesized, GSAD patients showed aberrant amygdala-related network and the short-term group CBT could normalize the abnormal hyper-connectivity of amygdala-prefrontal network. Furthermore, the changes of the left amygdala-dACC connectivity were associated with the clinical improvement (changes of the HAMA scores) in GSAD patients after treatment.

The most important finding of the current study is the abnormally elevated amygdala-dACC connectivity in GSAD patients, which is normalized along with clinical symptom improvement after group CBT. The dACC is densely and reciprocally connected with amygdala [35] which mostly sends projections to amygdala [36]. Moreover, joint activation pattern of amygdala and dACC have been identified in many neuroimaging studies on emotion [37, 38]. It has been previously reported that the dorsal–caudal regions of the ACC and mPFC are involved in the appraisal and expression of negative emotions, whereas the ventral–rostral portions of the ACC and mPFC play a regulatory role in negative emotion [39, 40]. The authors concluded that the amygdala-dmPFC/ACC connectivity is associated with “negative emotion generation” [39]. Abnormal amygdala-dACC connectivity has been previously reported in association with anxiety [13, 40]. Considering the above mentioned findings, and in conjunction with our results, the abnormal hyper-connectivity of amygdala-dACC connectivity could attribute an exaggerated significance to possible threat stimuli from the internal and the external environment, leading to a high level of anxiety in patients with GSAD. Besides, present study provide further evidence to support the 8 weeks of group CBT exert an effect on normalizing the amygdala–dACC connectivity along with the improvement of anxiety symptom. Since CBT could alleviate the predisposition for excessive fear and anxiety in response to environmental stimuli [41] by effective strategies such as relaxation training, in vivo exposure and social skills training, such therapy could relief the abnormal elevated connectivity between amygdala and dACC. Therefore, our finding not only provided robust evidence for the key role of the amygdala-dACC connectivity in GSAD pathogenesis, but also provide a potential biomarker to monitor the treatment effect of CBT in GSAD patients.

Besides, we also found that the short-term group CBT reduced the abnormally elevated connectivity of the left amygdala with the left dmPFC at baseline. In fact, the abnormal elevated amygdala-dmPFC connectivity has been reported during self-directed criticism in patients with generalized social phobia [42]. Both the dmPFC and the amygdala are regions associated with fear expression in response to stimuli from the external world and a positive amygdala-dmPFC connectivity was associated with hypervigilance [14]. Thus the observed impaired amygdala–dmPFC connectivity in our study may reflect the tendency to attribute an exaggerated significance to possible self-relevant stimuli from the processed threat cues in patients with GSAD [42, 43]. And the decreased coupling of these two regions after cognitive restructuring in group CBT could underlie this modulated knowledge of the self.

Beyond the above findings, we found decreased connectivity between the left amygdala and the right putamen in GSAD after treatment. The putamen is the one of the sectors of the striatum. The striatum has been proved to be the “emotion guarder”, which was an important terminal to receive the sensory and emotional information from the prefrontal areas [44]. Thus the decreased connectivity between the left amygdala and the right putamen in SAD may represent the short-term recovery process of the negtive emotions in social situations.

Another thing should be noted is that the altered connectivity only involved the left amygdala. This phenomenon could be explained by the abundantly reported lateralization of the human amygdala. The left amygdala was more often activated than the right amygdala, irrelevant to stimulus type [45]. Our finding of left amygdala-dominant brain networks at resting-state further suggested the asymmetric interhemispheric information transfer, in line with the left hemifield of amygdala advantage for fear processing in task-related studies [46].

The findings of the present study should be considered in the context of several limitations. First, the study design lacked re-test data of the healthy control group and a placebo or wait-list control. Thus, the neural and clinical findings cannot be causally attributed to group CBT and could be related to a number of plausible factors not related to the treatment, such as the natural course of the illness over the 8-week period and placebo/expectancy effects. Second, four of the GSAD patients were on SSRI medication at the time of study participation and the medications were reported to be ineffective in the treatment of this illness. However, the effects of medication should not be ignored in explaining the results. A recent study showed mediating effects of acute SSRI intake on prefrontal-amygdala effectivity connectivity [47], although the patients withdrawed medication at least two weeks before the baseline MRI scan in present study, our findings might be related to the medications. Third, although from previous studies we know that a group of 14 participants were sufficient to detect significant treatment effects measured with fMRI [48], the limited sample size (*n* = 15 GSAD subjects) increased the risk for false negatives and constrains the test for relationships between rsFC and treatment effects. These findings, however, may guide subsequent fMRI studies of treatment. Finally, there are a number of studies suggesting distinct functional connectivity patterns for the amygdala subregions such as the superficial and centromedial amygdala when choosing these subnuclei as seed [49, 50]. However, accurate delineation in the human brain is still under debate [51] and such approach has a major limitation as the amygdala is peculiarly susceptible to image distortion, normalization failure and draining vein effects [52]. Thus we chose a more conservative approach based on functional results of the whole amygdala region using the AAL template according to most human literature for investigation of the amygdala-related network.

## Conclusions

Our findings provide evidence that the short-term group CBT could attenuate the hyper-connectivity of amygdala with dACC and dmPFC along with the improvement of symptom severity during resting-state in patients with generalized social anxiety disorder. These findings not only provided robust evidence for the key role of the abnormal higher connectivity of prefrontal-amygdala in GSAD pathogenesis, but also provide a potential biomarker to monitor the treatment effect of CBT in GSAD patients.

## References

[1] American Psychiatric Association (2013). Diagnostic and Statistical Manual of Mental Disorders: DSM-V-TR.

[2] Ohayon MM, Schatzberg AF (2010). Social phobia and depression: prevalence and comorbidity. J Psychosom Res.

[3] Stein MB, Stein DJ (2008). Social anxiety disorder. Lancet.

[4] Adolphs R, Tranel D (2003). Amygdala damage impairs emotion recognition from scenes only when they contain facial expressions. Neuropsychologia.

[5] Amaral DG (2002). The primate amygdala and the neurobiology of social behavior: implications for understanding social anxiety. Biol Psychiatry.

[6] Adolphs R, Tranel D, Hamann S, Young AW, Calder AJ, Phelps EA (1999). Recognition of facial emotion in nine individuals with bilateral amygdala damage. Neuropsychologia.

[7] Lanteaume L, Khalfa S, Regis J, Marquis P, Chauvel P, Bartolomei F (2007). Emotion induction after direct intracerebral stimulations of human amygdala. Cereb Cortex.

[8] Irle E, Ruhleder M, Lange C, Seidler-Brandler U, Salzer S, Dechent P (2010). Reduced amygdalar and hippocampal size in adults with generalized social phobia. J Psychiatry Neurosci.

[9] Phan KL, Coccaro EF, Angstadt M, Kreger KJ, Mayberg HS, Liberzon I (2013). Corticolimbic brain reactivity to Social Signals of Threat Before and After Sertraline Treatment in Generalized Social Phobia. Biol Psychiatry.

[10] Phan KL, Fitzgerald DA, Nathan PJ, Tancer ME (2006). Association between amygdala hyperactivity to harsh faces and severity of social anxiety in generalized social phobia. Biol Psychiatry.

[11] Straube T, Mentzel HJ, Miltner WH (2005). Common and distinct brain activation to threat and safety signals in social phobia. Neuropsychobiology.

[12] Bruhl AB, Delsignore A, Komossa K, Weidt S (2014). Neuroimaging in social anxiety disorder-A meta-analytic review resulting in a new neurofunctional model. Neurosci Biobehav Rev.

[13] Demenescu LR, Kortekaas R, Cremers HR, Renken RJ, van Tol MJ, van der Wee NJ (2013). Amygdala activation and its functional connectivity during perception of emotional faces in social phobia and panic disorder. J Psychiatr Res.

[14] Straube T, Schmidt S, Weiss T, Mentzel HJ, Miltner WH (2009). Dynamic activation of the anterior cingulate cortex during anticipatory anxiety. Neuroimage.

[15] Hahn A, Stein P, Windischberger C, Weissenbacher A, Spindelegger C, Moser E (2011). Reduced resting-state functional connectivity between amygdala and orbitofrontal cortex in social anxiety disorder. Neuroimage.

[16] Carlson JM, Greenberg T, Rubin D, Mujica-Parodi LR (2011). Feeling anxious: anticipatory amygdalo-insular response predicts the feeling of anxious anticipation. Soc Cogn Affect Neurosci.

[17] Linden DE (2006). How psychotherapy changes the brain – the contribution of functional neuroimaging. Mol Psychiatry.

[18] Fox MD, Raichle ME (2007). Spontaneous fluctuations in brain activity observed with functional magnetic resonance imaging. Nat Rev Neurosci.

[19] Greicius MD, Krasnow B, Reiss AL, Menon V (2003). Functional connectivity in the resting brain: a network analysis of the default mode hypothesis. Proc Natl Acad Sci U S A.

[20] Dodhia S, Hosanagar A, Fitzgerald DA, Labuschagne I, Wood AG, Nathan PJ (2014). Modulation of resting-state amygdala-frontal functional connectivity by oxytocin in generalized social anxiety disorder. Neuropsychopharmacology.

[21] Heimberg RG (2002). Cognitive-behavioral therapy for social anxiety disorder: current status and future directions. Biol Psychiatry.

[22] Canton J, Scott KM, Glue P (2012). Optimal treatment of social phobia: systematic review and meta-analysis. Neuropsychiatr Dis Treat.

[23] Fedoroff IC, Taylor S (2001). Psychological and pharmacological treatments of social phobia: a meta-analysis. J Clin Psychopharmacol.

[24] Klumpp H, Keutmann MK, Fitzgerald DA, Shankman SA, Phan KL (2014). Resting state amygdala-prefrontal connectivity predicts symptom change after cognitive behavioral therapy in generalized social anxiety disorder. Biol Mood Anxiety Disord.

[25] Hofmann SG, Otto MW (2008). Cognitive Behavioral Therapy for Social Anxiety Disorder: Evidence-Based and Disorder-Specific Treatment Techniques.

[26] First MB SRL, Gibbon M WJ. Structured Clinical Interview for the DSM-IV Axis I Disorders: SCID-I/P, Version 2.0. Biometrics Research Department, New York State Psychiatric Institute 1997.

[27] Heimberg RG, Horner KJ, Juster HR, Safren SA, Brown EJ, Schneier FR (1999). Psychometric properties of the Liebowitz Social Anxiety Scale. Psychol Med.

[28] Rytwinski NK, Fresco DM, Heimberg RG, Coles ME, Liebowitz MR, Cissell S (2009). Screening for social anxiety disorder with the self-report version of the Liebowitz Social Anxiety Scale. Depress Anxiety.

[29] Hope DA, Heimberg RG, Turk CL (2006). Managing Social Anxiety: A Cognitive-Behavioral Therapy Approach Therapist Guide.

[30] Behzadi Y, Restom K, Liau J, Liu TT (2007). A component based noise correction method (CompCor) for BOLD and perfusion based fMRI. Neuroimage.

[31] Gotts SJ, Saad ZS, Jo HJ, Wallace GL, Cox RW, Martin A (2013). The perils of global signal regression for group comparisons: a case study of Autism Spectrum Disorders. Front Hum Neurosci.

[32] Biswal B, Yetkin FZ, Haughton VM, Hyde JS (1995). Functional connectivity in the motor cortex of resting human brain using echo-planar MRI. Magn Reson Med.

[33] Tzourio-Mazoyer N, Landeau B, Papathanassiou D, Crivello F, Etard O, Delcroix N (2002). Automated anatomical labeling of activations in SPM using a macroscopic anatomical parcellation of the MNI MRI single-subject brain. Neuroimage.

[34] Power JD, Barnes KA, Snyder AZ, Schlaggar BL, Petersen SE (2013). Steps toward optimizing motion artifact removal in functional connectivity MRI; a reply to Carp. Neuroimage.

[35] Ongur D, Price JL (2000). The organization of networks within the orbital and medial prefrontal cortex of rats, monkeys and humans. Cereb Cortex.

[36] Ghashghaei HT, Hilgetag CC, Barbas H (2007). Sequence of information processing for emotions based on the anatomic dialogue between prefrontal cortex and amygdala. Neuroimage.

[37] Kober H, Barrett LF, Joseph J, Bliss-Moreau E, Lindquist K, Wager TD (2008). Functional grouping and cortical-subcortical interactions in emotion: a meta-analysis of neuroimaging studies. Neuroimage.

[38] Seeley WW, Menon V, Schatzberg AF, Keller J, Glover GH, Kenna H (2007). Dissociable intrinsic connectivity networks for salience processing and executive control. J Neurosci.

[39] Etkin A, Egner T, Kalisch R (2011). Emotional processing in anterior cingulate and medial prefrontal cortex. Trends Cogn Sci.

[40] Etkin A, Wager TD (2007). Functional neuroimaging of anxiety: a meta-analysis of emotional processing in PTSD, social anxiety disorder, and specific phobia. Am J Psychiatry.

[41] Bogels SM, Mansell W (2004). Attention processes in the maintenance and treatment of social phobia: hypervigilance, avoidance and self-focused attention. Clin Psychol Rev.

[42] Blair K, Geraci M, Devido J, McCaffrey D, Chen G, Vythilingam M (2008). Neural response to self- and other referential praise and criticism in generalized social phobia. Arch Gen Psychiatry.

[43] Clark DM, McManus F (2002). Information processing in social phobia. Biol Psychiatry.

[44] Friedman A, Homma D, Gibb LG, Amemori K, Rubin SJ, Hood AS (2015). A Corticostriatal Path Targeting Striosomes Controls Decision-Making under Conflict. Cell.

[45] Baas D, Aleman A, Kahn RS (2004). Lateralization of amygdala activation: a systematic review of functional neuroimaging studies. Brain Res Brain Res Rev.

[46] Siman-Tov T, Papo D, Gadoth N, Schonberg T, Mendelsohn A, Perry D (2009). Mind your left: spatial bias in subcortical fear processing. J Cogn Neurosci.

[47] Sladky R, Spies M, Hoffmann A, Kranz G, Hummer A, Gryglewski G (2015). (S)-citalopram influences amygdala modulation in healthy subjects: a randomized placebo-controlled double-blind fMRI study using dynamic causal modeling. Neuroimage.

[48] Klumpp H, Fitzgerald DA, Phan KL (2013). Neural predictors and mechanisms of cognitive behavioral therapy on threat processing in social anxiety disorder. Prog Neuropsychopharmacol Biol Psychiatry.

[49] Blackford JU, Clauss JA, Avery SN, Cowan RL, Benningfield MM, VanDerKlok RM (2014). Amygdala-cingulate intrinsic connectivity is associated with degree of social inhibition. Biol Psychol.

[50] Etkin A, Prater KE, Schatzberg AF, Menon V, Greicius MD (2009). Disrupted amygdalar subregion functional connectivity and evidence of a compensatory network in generalized anxiety disorder. Arch Gen Psychiatry.

[51] Amunts K, Kedo O, Kindler M, Pieperhoff P, Mohlberg H, Shah NJ (2005). Cytoarchitectonic mapping of the human amygdala, hippocampal region and entorhinal cortex: intersubject variability and probability maps. Anat Embryol (Berl).

[52] Robinson S, Windischberger C, Rauscher A, Moser E (2004). Optimized 3T EPI of the amygdalae. Neuroimage.

